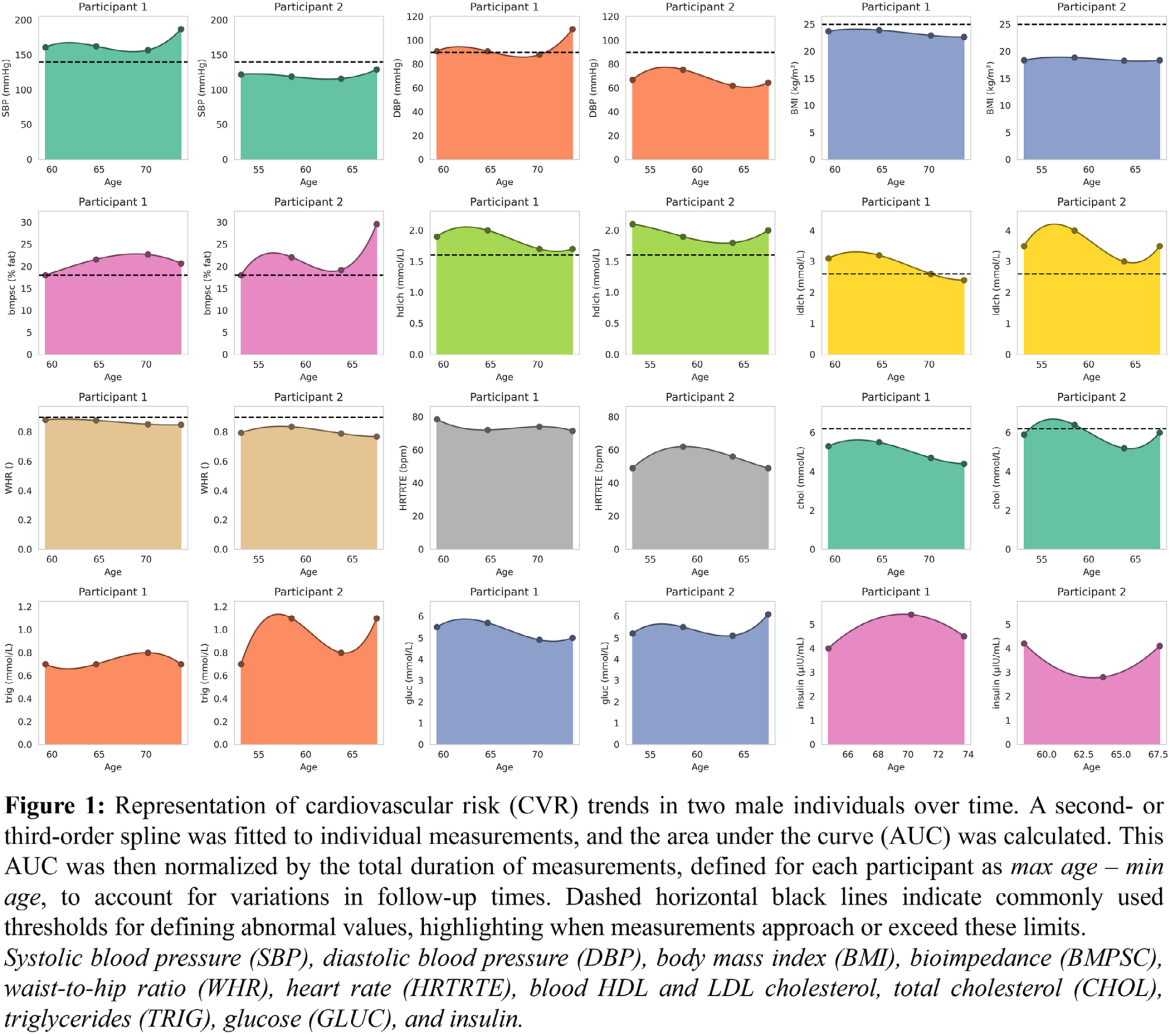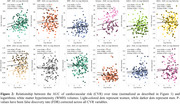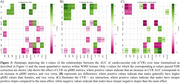# Longitudinal cardiovascular risks and their impact on white matter hyperintensities

**DOI:** 10.1002/alz70862_110114

**Published:** 2025-12-23

**Authors:** Aurelie Bussy, Camille Cathala, Ferath Kherif, Antoine Lutti, Bogdan Draganski

**Affiliations:** ^1^ Inselspital, Bern, Bern Switzerland; ^2^ EPFL, Lausanne, Vaud Switzerland; ^3^ Centre Hospitalier Universitaire Vaudois, Lausanne, Vaud Switzerland; ^4^ CHUV, Lausanne, Vaud Switzerland

## Abstract

**Background:**

Cerebral small vessel disease, a major cause of aging‐related stroke and cognitive decline, often manifests as white matter hyperintensities (WMH) on MRI1–3. WMHs vary in location and tissue microstructure, reflecting underlying pathology such as demyelination and axonal loss. Cardiovascular risk factors (CVR), including elevated systolic blood pressure (SBP) and heart rate, are linked to WMH development4. This study explores the relationship between longitudinal CVR and WMH characteristics, including quantitative MRI (qMRI) analyses of microstructural alterations.

**Method:**

Data were obtained from 539 participants in the BrainLaus study (CoLaus|PsycoLaus cohort, Lausanne, Switzerland). 153 participants (86 females) were selected on the basis of at least three CVR measurements, with a mean follow‐up duration of 14.45 years. CVR factors included SBP, diastolic blood pressure (DBP), body mass index (BMI), bioimpedance, waist‐to‐hip ratio (WHR), heart rate, cholesterol (HDL, LDL, total), triglycerides, glucose, and insulin. Imaging was performed on a 3T Magnetom Prisma using FLAIR, T1‐weighted MPRAGE, multi‐echo FLASH, and DWI. WMH were segmented using a ResUNet‐based algorithm5, and quantitative maps (MTsat, R2*, R1) were generated6. CVR over time was modeled using splines, and area under the curve (AUC) was calculated for each participant, normalized by total duration. Statistical analysis included linear models with sex interaction terms, corrected for false discovery rate (FDR).

**Result:**

Figure 1 shows CVR AUC over time, highlighting inter‐individual variation. Figure 2 demonstrates that increased CVR (SBP, LDL, glucose, WHR, bioimpedance, total cholesterol) was associated with larger WMH volumes, with stronger effects in women for SBP, LDL, total cholesterol, and triglycerides. Figure 3 shows that CVR impacts WMH microstructure, with cholesterol‐related risk factors linked to higher water content (MD, ISOVF) in women, and hypertension/heart rate linked to lower myelin content (MT, R1) in men. Men showed increased g‐ratio indicating myelin thickness reduction with higher CVR.

**Conclusion:**

The impact of CVR on WMH differs by sex, with women showing greater effects on water content and men more on myelin integrity. These findings suggest that sex‐specific approaches may be needed for understanding and targeting cardiovascular risk factors to preserve white matter health and prevent cognitive decline.